# Patients’ and carers’ views on research priorities in prehabilitation for cancer surgery

**DOI:** 10.1007/s00520-024-08585-1

**Published:** 2024-05-24

**Authors:** Jennifer Vu, Cherry Koh, Michael Solomon, Kilian Brown, Sascha Karunaratne, Ruby Cole, Phillippa Smith, Pratik Raichurkar, Linda Denehy, Bernhard Riedel, Prehabilitation Expert Collaborative, Jonathan Allen, Kevin Ancog, Eva Angenete, Nabila Ansari, Fabio Ausania, Anna Beaumont, Christian Beilstein, Frederik Berrevoet, Ianthe Boden, Bert Bongers, Kimberley Bostock, Janine Bothe, Birgitte Brandstrup, Louise Brennan, Sorrel Burden, Crystal Burgess, Elaine Burns, Francesco Carli, Vinicius Cavalheri, Wim Ceelen, Tyler Chesney, David Clark, Kari Clifford, Kelcie Cole, Thomas Collyer, Rob Copeland, Roland Croner, Jess Crowe, Ian Daniels, Gerard Danjoux, June Davis, Caitlin Davis, Mayke de Klerk, Tina Decorte, Jan Willem Dekker, Andreas Denys, Liesbeth Desender, Pieter Dries, Declan Dunne, Lara Edbrooke, Linda Edgar, Sabry Eissa, Dominique Engel, James Ephraums, Martyn Evans, Rhonda Farrell, Alice Finch, Aisling Fleury, Patrice Forget, Nader Francis, Frank Frizelle, Walter Frontera, Karen Geboes, Hugh Giddings, Chris Gillespie, Chelsia Gillis, Olivier Glehen, Varsha Gorey, Catherine Granger, Diana Greenfield, Ben Griffiths, Chloe Grimmett, Claire Hackett, Travis Hall, Julie Hallet, Craig Harris, Sophie Hatcher, Lizza Hendriks, Mendy Hermans, Carl Ilyas, Hilmy Ismail, John Jenkins, Wilson Jiang, Charlotte Johnstone, Andreas Karakatsanis, Simarjit Kaur, Michael Kelly, Joost Klaase, Dorian Kršul, Scott Leslie, Jenelle Loeliger, Marie-Louise Lydrup, Andrea Maier, Piotr Major, Preet Makker, Christopher Mantyh, Stuart McCluskey, Laura McGarrity, Jayson Moloney, Isacco Montroni, Brendan Moran, Paul Morris, Susan Moug, Rajeswari Ms, Sandra Murdoch, Anna Myers, Kheng-Seong Ng, Per J. Nilsson, Peter Noordzij, Mike O’Connor, Gianluca Pellino, Shannon Philp, Marc Pocard, Zudin Puthucheary, Emma Putrus, Aaron Quyn, Thomas Read, William Ricketts, Harm Rutten, Charissa Sabajo, Rawand Salihi, Tarik Sammour, Charbel Sandroussi, Daniel Santa Mina, Stefan Saric, Raquel Sebio, Doruk Seyfi, Favil Singh, Gerrit Slooter, Neil Smart, Lissa Spencer, Paul Sutton, Hao Ern Tan, David Ten Cate, Akif Turna, Elke Van Daele, Adinda van den Berg, Charlotte van Kessel, Gabrielle van Ramshorst, Emiel Verdaasdonk, Chris Wakeman, Malcolm West, James Wheeler, Duminda Wijeysundera, Hideaki Yano, Daniel Steffens

**Affiliations:** 1 Surgical Outcomes Research Centre (SOuRCe), Royal Prince Alfred Hospital (RPAH), Missenden Road, PO Box M157, Sydney, NSW 2050 Australia; 2 Faculty of Medicine and Health, Central Clinical School, The University of Sydney, Sydney, Australia; 3 Institute of Academic Surgery (IAS), Royal Prince Alfred Hospital (RPAH), Sydney, Australia; 4 Department of Colorectal Surgery, Royal Prince Alfred Hospital (RPAH), Sydney, Australia; 5 Peter MacCallum Cancer Centre, Melbourne, VIC Australia; 6 Department of Physiotherapy, The University of Melbourne, Melbourne, VIC Australia; 7 The Sir Peter MacCallum Department of Oncology, University of Melbourne, Melbourne, VIC Australia; 8 Department of Critical Care, The University of Melbourne, Melbourne, VIC Australia

**Keywords:** Cancer, Prehabilitation, Surgery, Preoperative, Research priorities, Outcomes

## Abstract

**Introduction:**

The views of patients and carers are important for the development of research priorities. This study aimed to determine and compare the top research priorities of cancer patients and carers with those of multidisciplinary clinicians with expertise in prehabilitation.

**Materials and methods:**

This cross-sectional study surveyed patients recovering from cancer surgery at a major tertiary hospital in Sydney, Australia, and/or their carers between March and July 2023. Consenting patients and carers were provided a list of research priorities according to clinicians with expertise in prehabilitation, as determined in a recent International Delphi study. Participants were asked to rate the importance of each research priority using a 5-item Likert scale (ranging from 1 = very high research priority to 5 = very low research priority).

**Results:**

A total of 101 patients and 50 carers participated in this study. Four areas were identified as research priorities, achieving consensus of highest importance (> 70% rated as “high” or “very high” priority) by patients, carers, and clinical experts. These were “optimal composition of prehabilitation programs” (77% vs. 82% vs. 88%), “effect of prehabilitation on surgical outcomes” (85% vs. 90% vs. 95%), “effect of prehabilitation on functional outcomes” (83% vs. 86% vs. 79%), and “effect of prehabilitation on patient reported outcomes” (78% vs. 84% vs. 79%). Priorities that did not reach consensus of high importance by patients despite reaching consensus of highest importance by experts included “identifying populations most likely to benefit from prehabilitation” (70% vs. 76% vs. 90%) and “defining prehabilitation core outcome measures” (66% vs. 74% vs. 87%). “Prehabilitation during neoadjuvant therapies” reached consensus of high importance by patients but not by experts or carers (81% vs. 68% vs. 69%).

**Conclusion:**

This study delineated the primary prehabilitation research priorities as determined by patients and carers, against those previously identified by clinicians with expertise in prehabilitation. It is recommended that subsequent high-quality research and resource allocation be directed towards these highlighted areas of importance.

**Supplementary Information:**

The online version contains supplementary material available at 10.1007/s00520-024-08585-1.

## Introduction

Prehabilitation is the preparation of patients for surgery with the aim to enhance resilience and functional capacity for optimal postoperative recovery and expedited return to baseline daily activities [1–4]. It encompasses the use of physical, nutritional, and psychosocial interventions to address modifiable risk factors and thereby improve preoperative functional capacity [5, 6]. Previous studies have shown the same multidisciplinary program utilized as prehabilitation (before surgery) instead of rehabilitation (after surgery) leads to higher rates of recovery to baseline measurements at 8 weeks postoperatively [7]. Patients with lower baseline fitness have been reported to demonstrate the most benefit [8, 9]. The evidence for improved outcomes by prehabilitation is growing with two large overviews of systematic reviews in abdominal and lung cancer surgery that include 55 and 30 systematic reviews respectively reporting positive results [10, 11]. Studies comparing prehabilitation to no prehabilitation have reported an approximately 50% reduction in postoperative complications and up to a 3-day reduction in length of hospital stay in favor of the prehabilitation group [12–15]. Furthermore, Trépanier and colleagues [16] reported an improved 5-year disease-free survival of patients with stage III colorectal cancer in the prehabilitation group (73.4%) compared to the control group (50.9%; *p* = 0.04).

A major limitation of the current literature is the heterogeneity among published trials [17]. Previous prehabilitation trials generally included small sample sizes and assessed a variety of interventions on non-standardized postoperative outcomes. This limits the comparability and generalizability of these data, making it difficult to form recommendations on a larger scale. Furthermore, the most suitable candidate groups for prehabilitation have not been defined. By identifying research priorities, we anticipate increased collaboration between clinicians with expertise in prehabilitation (experts) at an international level to establish universally applicable outcome measures, interventions, and target patient groups.

A recent study by Raichurkar et al. [18] has taken the first step by utilizing the Delphi methodology to identify the consensus of prehabilitation research priorities according to an international, multidisciplinary group of experts [18]. A total of 12 priorities reached consensus of high importance including “identifying populations most likely to benefit from prehabilitation,” “optimal composition of prehabilitation programs,” and “effect of prehabilitation on surgical outcomes.” This information facilitates comparability of future prehabilitation research output to enhance the evidence base. However, a key limitation of this study was the lack of input from cancer patients and/or their carers, thereby overlooking the importance of the usefulness of a prehabilitation program from a patient perspective.

The primary aim of this cross-sectional cohort study was to gain consensus on the top prehabilitation research priorities according to cancer patients and their carers. The secondary aim was to compare the top prehabilitation research priorities of cancer patients and carers with those of experts in prehabilitation. The results of this study are hypothesized to guide the development of future prehabilitation trials in the cancer surgery setting and facilitate meaningful allocations of resources.

## Methods

### Study design and setting

This cross-sectional study surveyed patients in the postoperative period following cancer surgery at a major tertiary hospital in Sydney, Australia, and/or their carers between March and July 2023. This manuscript followed the reporting recommendations from the STROBE statement [19]. Ethics and Governance authorization was obtained from the Sydney Local Health District Human Ethics Committee (HREC X21-0361) and conducted in accordance with the World Medical Association Declaration of Helsinki (2013).

### Participant selection and recruitment

Patients were eligible if aged 18 years or over and in the postoperative period following thoracic, upper gastrointestinal, or colorectal cancer surgery at Royal Prince Alfred Hospital in Sydney, Australia. These tumor groups were chosen because (i) these are among the most prevalent tumor types in our setting, ensuring adequate sample size; (ii) preoperative care for these patients is a high priority for our institution; (iii) despite the tumor differences, these patients have a somewhat similar postoperative trajectory, allowing for meaningful comparisons; (iv) prior research has identified a need for more evidence to guide preoperative management in these populations; and (v) these groups had the most consistent surgical volume during our recruitment period, enhancing feasibility. Other tumor groups remain important and will be included in future studies. Patients and/or carers were excluded if they had inadequate English language competency to complete the survey. Participants were identified and recruited prospectively at Royal Prince Alfred Hospital. This involved the complete sampling of eligible participants throughout March to July 2023. Consecutive patients were approached in-person on the wards during their post-operative period. Collaboration with treating teams occurred to identify which days were most appropriate to approach potential participants for discussion about the study, for example, following mobilization after the surgery, and when their carers were most likely to be there, for example, in the afternoons.

Carers, or caregivers, were invited to participate if present with the patient on the ward at the time of approach by the study investigator. A recent literature review identified informal caregivers to be individuals with a strong personal connection to the person with cancer, for example, a spouse/partner, family member, or friend [20]. Caregivers provide a broad range of assistance with most aspects of daily life, including but not limited to cooking, dressing, and transport to healthcare appointments. Any visitor accompanying a patient with cancer fitting this description was invited to participate.

### Preparatory steps

A Delphi process was conducted to establish consensus on key prehabilitation research priorities according to a multidisciplinary group of clinicians with expertise in prehabilitation. This is now published [18]. In brief, the Delphi methodology was implemented over three rounds of surveys distributed to 165 experts, including surgeons, anesthesiologists, physiotherapists, dieticians, nurses, and academic researchers across four continents, including Australia, Asia, Europe, and North America. A total of 75 unique prehabilitation research priorities were identified in the first round of the Delphi process. Over the following two successive survey rounds, a total of 12 research priorities reached consensus with the ranking of “highest importance.” These top 12 research priorities were then utilized in this study that surveyed patients and their carers.

### Survey design

The study consisted of a single survey distributed to inpatients recovering from cancer surgery and/or their carers. This was within the first 30 days of their postoperative period and irrespective of participation in prehabilitation before the surgery. The survey contained three sections:

- **Section 1** (demographics): Age, sex, medical history including type of cancer, surgical procedure, other treatment received, and if applicable, previous prehabilitation exposure including type and duration of prehabilitation and type of health professionals involved in their prehabilitation (i.e., anesthesiologist, physiotherapist, surgeon, nurse, psychologist, dietician).
- **Section 2** (research priority ratings): The list of research priorities previously established by the experts in prehabilitation through the Delphi process was provided. Patients and carers were asked to rate the importance of each priority using a 5-item Likert scale (1 = very high research priority; 2 = high; 3 = moderate; 4 = low; 5 = very low research priority). This was the same scale used by the experts. Consensus was considered if > 70% of participants indicated agreement on the research priority.
- **Section 3** (open-ended questions): The first question invited patients and carers to provide feedback on the provided research priorities and the second question invited patients and carers to suggest additional research priorities they believed should be included.

### Data analysis

All survey data were collected and managed using REDCap (Vanderbilt University, Nashville, TN, USA) electronic data capture tools hosted at the Sydney Local Health District [21, 22]. Descriptive statistics and frequency distributions were calculated to describe the responses of participants and were displayed as frequency (percentage) and median (IQR) according to order of importance (i.e., very high priority to very low priority). Differences in prehabilitation research priorities between patients, carers, and experts were determined by the chi-squared test and Kruskal–Wallis test. All analyses were performed using SPSS version 28 with a significance level set at *p* < 0.05.

## Results

### Participant flow

Of the 131 patients screened and approached, 101 (77.1%) patients participated in the study. Reasons for non-participation included inadequate English language competency to complete the survey (*n* = 23, 17.6%) and not interested (*n* = 7, 5.3%). Of the 55 carers present with an eligible and consenting patient at the time of approach by a study investigator, 50 (90.9%) carers participated. This included carers accompanying patients with inadequate English language competency (*n* = 9, 16.4%) and one patient who was too unwell to complete the survey (*n* = 1, 1.8%).

### Characteristics of the included participants

Detailed characteristics of the included cohort are found in Table 1. Patients had a mean (standard deviation, SD) age of 56.6 (13.6) years and included 46 (45.5%) males. Carers had a mean (SD) age of 53.2 (14.6) years and included 26 (52.0%) males. A total of 66 (65.3%) patients underwent colorectal cancer surgery, 21 (20.8%) upper gastrointestinal cancer surgery, and 14 (13.9%) thoracic cancer surgery; 58 (57.4%) patients underwent neoadjuvant chemotherapy. There were 49 (48.5%) patients who engaged in a prehabilitation program.

**Table 1:** Expert, patient, and carer demographics

Characteristics	Patients (*n* = 101)	Carers (*n* = 50)	Clinicians with expertise in prehabilitation (experts) (*n* = 121)
Sex			
Male Female Not specified	46 (45.5%) 55 (54.5%) 0 (0.0%)	26 (52.0%) 24 (48.0%) 0 (0.0%)	68 (56.2%) 37 (30.6%) 16 (13.2%)
Age, years			
18–40 41–60 > 60 Not specified	13 (12.9%) 46 (45.5%) 41 (40.6%) 1 (1.0%)	12 (24.0%) 20 (40.0%) 17 (34.0%) 1 (2.0%)	31 (25.6%) 62 (51.2%) 12 (9.9%) 16 (13.2%)
Body mass index			
< 18.5 kg/m^2^ 18.5–24.9 kg/m^2^ 25.0–29.9 kg/m^2^ ≥ 30.0 kg/m^2^	2 (2.0%) 49 (48.5%) 32 (31.7%) 18 (17.8%)	*N/A*	*N/A*
Type of cancer			
Colorectal Upper gastrointestinal Thoracic	66 (65.3%) 21 (20.8%) 14 (13.9%)	*N/A*	*N/A*
Surgical procedure			
Cytoreduction + / − HIPEC Pelvic exenteration Upper gastrointestinal resection (e.g., hepatectomy) Lobectomy or pneumonectomy Bowel resection Localized tumor resection (thoracic) Localized tumor resection (abdominal)	32 (31.7%) 26 (25.7%) 19 (18.8%) 8 (7.9%) 5 (5.0%) 6 (5.9%) 5 (5.0%)	*N/A*	*N/A*
Neoadjuvant treatment			
Chemotherapy only Chemotherapy and radiotherapy None	33 (32.7%) 25 (24.8%) 43 (42.6%)	*N/A*	*N/A*
Prehabilitation provided			
No prehabilitation Prehabilitation	52 (51.5%) 49 (48.5%)	*N/A*	*N/A*
Type of prehabilitation			
Physical exercise Nutrition support Psychological support	24 (49.0%) 32 (65.3%) 17 (34.7%)		
Professionals involved in prehabilitation			
Physiotherapist Dietitian Psychologist	22 (44.9%) 30 (61.2%) 19 (38.8%)	*N/A*	*N/A*
Duration of prehabilitation			
0–4 weeks 1–6 months > 6 months	36 (73.5%) 11 (22.4%) 1 (2.0%)		

Data presented as frequency (percentage)

### Patient and carer views on research priorities

The top three research priorities that reached consensus of highest importance (> 70% rated as “high or very high research priority”) by patients were “effect of prehabilitation on surgical outcomes” (85%, median = 1.0; IQR = 1.0 to 2.0), “effect of prehabilitation on functional outcomes” (83%, median = 2.0; IQR = 1.0 to 2.0), and “prehabilitation during neoadjuvant therapies” (81%, median = 2.0; IQR = 1.0 to 2.0). Priority ratings for “prehabilitation during neoadjuvant therapies” differed significantly between patients who received neoadjuvant therapy and those who did not. Among patients who underwent neoadjuvant treatment, 91.3% (53 out of 58) rated this item as “high” or “very high” importance, compared to 69.0% (29 out of 42) of patients who did not receive neoadjuvant therapy (*p* = 0.004).

**Fig. 1:**
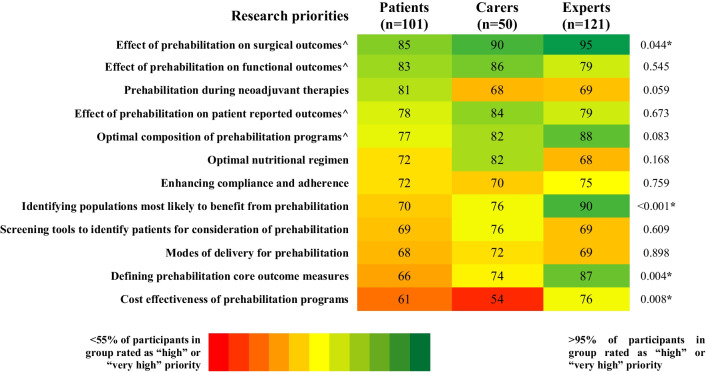
Prehabilitation research priorities according to experts’, patients’, and carers’ views. Data presented as percentage. Research priorities listed in descending order from highest priority to lowest priority according to patient consensus. Research priority rating scale: 1 = very high priority, 2 = high priority, 3 = moderate priority, 4 = low priority, or 5 = very low priority. High research priority determined by the number of experts, patients, and carers that rated 1 (very high priority) or 2 (high priority) in the research priority rating scale. Research priority reached consensus of high importance by a group if rated as 1 (very high) or 2 (high) by > 70% of participants in the group. Color scale: red (less than 55% of participants in group rated 1 (very high priority) or 2 (high priority) in the research priority rating scale) to dark green (more than 95% of participants in group rated 1 (very high priority) or 2 (high priority) in the research priority rating scale), with yellow at midpoint (75% of participants in group rated 1 (very high priority) or 2 (high priority) in the research priority rating scale). ^Research priority reached consensus of high importance by all groups. *Significant difference between ratings across expert, patient, and carer groups (*p* < 0.05)

The top three research priorities that reached consensus of highest importance by carers were “effect of prehabilitation on surgical outcomes” (90%, median = 1.0; IQR = 1.0 to 2.0), “effect of prehabilitation on functional outcomes” (86%, median = 2.0; IQR = 1.0 to 2.0), and “effect of prehabilitation on patient reported outcomes” (84%, median = 1.0; IQR = 1.0 to 2.0), two of these three being the same as patients. The complete list of research priorities and a comparison between groups are shown in Fig. 1 and Supplementary Table 1.

Patients who engaged in prehabilitation generally rated all priorities higher than patients who did not engage in prehabilitation however variation between priorities followed a similar trend. “Effect of prehabilitation on surgical outcomes” and “prehabilitation during neoadjuvant therapies” were among the top three priorities in both patient subgroups. The key differences between these subgroups were that “effectiveness of prehabilitation on patient-reported outcomes,” “optimal composition of prehabilitation programs,” and “identifying populations most likely to benefit from prehabilitation” reached consensus of high importance by patients who engaged in prehabilitation but not by those who did not engage in prehabilitation. This reflects differences in familiarity with prehabilitation attributable to prior experience. The differences between the patient subgroups are shown in Table 2.

**Table 2:** Comparison of patient research priorities according to engagement in prehabilitation

	Rated as high or very high priority		
Research priorities	Prehabilitation (*n* = 49)	No prehabilitation (*n* = 52)	*p*-value
Effect of prehabilitation on surgical outcomes	46 (94)^	40 (77)^	0.017*
Effect of prehabilitation on patient reported outcomes	43 (88)^	36 (69)	0.024*
Prehabilitation during neoadjuvant therapies	42 (86)^	40 (77)^	0.343
Optimal composition of prehabilitation programs	42 (86)^	36 (69)	0.048*
Effect of prehabilitation on functional outcomes	40 (82)^	44 (85)^	0.689
Optimal nutritional regimen	40 (82)^	33 (63)	0.041
Identifying populations most likely to benefit from prehabilitation	39 (80)^	32 (62)	0.047*
Enhancing compliance and adherence	37 (76)^	36 (69)	0.481
Screening tools to identify patients for consideration of prehabilitation	36 (73)^	34 (65)	0.379
Modes of delivery for prehabilitation	36 (73)^	33 (63)	0.28
Defining prehabilitation core outcome measures	36 (73)^	31 (60)	0.277
Cost effectiveness of prehabilitation programs	34 (69)	28 (54)	0.109

Data presented as frequency (percentage). Prehabilitation, patients who engaged in prehabilitation. No prehabilitation, patients who did not engage in prehabilitation. Research priorities listed in descending order from highest priority to lowest priority according to consensus of patients who engaged in prehabilitation. Research priority rating scale: 1 = very high priority, 2 = high priority, 3 = moderate priority, 4 = low priority, or 5 = very low priority. High research priority determined by the number of patients that rated 1 (very high priority) or 2 (high priority) in the research priority rating scale. Research priority reached consensus of high importance if rated as 1 (very high) or 2 (high) by > 70% of participants in the group. ^Research priority reached consensus of high importance by group. *Significant difference in ratings between prehabilitation and no prehabilitation groups (*p* < 0.05)

All patients and carers were invited to suggest additional research priorities not covered in the predefined list of 12 priorities, of which 21 out of 101 patients (21.8%) suggested additional research priorities. Multiple suggestions pertained to the effect of education, empathy, and/or patient involvement on outcomes (*n* = 5, 4.95%) or the effect of financial or residential situation/support on outcomes (*n* = 3, 3.0%). Other suggestions included incentivization to assist with self-discipline for prehabilitation, best psychology interventions and other non-medical interventions, how to ensure physiotherapists in remote areas are appropriately experienced to provide prehabilitation, and factoring in compound illnesses (e.g., muscular dystrophy). Seven out of 50 carers (14.0%) suggested additional research priorities, and these included better communication pre- and post-operatively to manage expectations and improve outcomes (*n* = 4, 8.0%), streamlined delivery modes (*n* = 1, 2.0%), psychology interventions to prepare patients for unexpected outcomes (*n* = 1, 2.0%), and connecting cancer patients with each other to improve mental health before surgery (*n* = 1. 2.0%).

## Discussion

### Similarities with the consensus achieved by clinicians with expertise in prehabilitation

Four out of the top 12 research priorities established by a Delphi process with clinicians with expertise in prehabilitation [18] achieved agreement of high importance by all three groups — clinician experts, patients, and carers. These were “optimal composition of prehabilitation programs,” “effect of prehabilitation on surgical outcomes,” “effect of prehabilitation on functional outcomes,” and “effect of prehabilitation on patient reported outcomes.”

These findings support the direction of prehabilitation research towards enhancing the evidence base in these areas. Previous studies have sought to address these areas; however, the strength of evidence remains relatively weak [1–4, 23–25]. The pursuit of future high-quality trials to advance these areas is supported by the findings of this study. Future research on the optimal composition of prehabilitation programs for the different outcomes may provide further benefit.

### Differences to the views of clinicians with expertise in prehabilitation

“Prehabilitation during neoadjuvant therapies” was among the top three research priorities of patients; however, it did not reach consensus of high importance by experts nor carers. This may reflect the difference in lived experience with neoadjuvant therapies between the groups. Chemotherapy is well known to cause unpleasant side effects including nerve pain, fatigue, reduced functional capacity, and vomiting [26], all of which could adversely impact or benefit from prehabilitation. However, 81% of patients rated this priority as “high” or “very high” priority despite only 57.4% having undergone neoadjuvant chemotherapy, suggesting the possibility of other contributing factors. Despite the known adverse effects, it is possible that experts may have placed greater emphasis on exploring less researched areas, given the existing strong evidence base in this domain. In contrast, patients’ ratings likely reflect the significant real-world impact of neoadjuvant therapies on their daily lives and overall wellbeing. This discrepancy highlights the importance of considering both clinical expertise and patient perspectives when setting research priorities. Our findings support future research towards determining the safety and potential benefits of prehabilitation during neoadjuvant therapies.

Three research priorities did not reach consensus of high importance by patients despite reaching consensus of high importance by experts and these were “identifying populations most likely to benefit from prehabilitation,” “defining prehabilitation core outcome measures,” and “cost effectiveness of prehabilitation programs.” These priorities pertain more to the practicalities of prehabilitation research and implementation. The top three priorities of patients as mentioned above suggest that patients tended to place higher importance on research associated with improved experiences and outcomes, and lower importance on practicalities. That said, more than half of patients felt that the research priority was of either “high” or “very high” research importance in all 12 research areas identified by the clinician expert group using a prior Delphi process. Importantly, the survey responses to these priorities may potentially be influenced by participants’ socioeconomic circumstances. The difference in responses between patients and experts may reflect differences in experience with health systems between the patient and expert groups and should be considered in future research. Additionally, patients voiced that everyone should be offered prehabilitation, not just those most likely to benefit.

These results support the direction of future prehabilitation research towards establishing the effectiveness of prehabilitation on patient outcomes and experiences. Research focussing on the practicalities of prehabilitation may still be valued by patients but perhaps only if required to measure the benefits of prehabilitation on patient outcomes, until these benefits become well established. The carer consensus further supports this premise.

### Additional trends in patient and carer research priorities

It is notable but understandable that “cost effectiveness of prehabilitation programs” was furthest from reaching consensus in both the patient and carer groups (61% vs. 54%). It is undeniable that cost is a major barrier for implementation of new models of care, however perhaps costs (system or personal) would not deter participation; however, this question was not directly addressed in this survey. However, financial toxicity is reported as an issue for patients with cancer [27]. At the Royal Prince Alfred Hospital, prehabilitation is conducted within a research framework. The prehabilitation team was running a number of trials funded by major Australian grants, including the National Health and Medical Research Council (NHMRC) and Medical Research Future Fund (MRFF), concurrently and independently of this study. Participation in these trials was completely voluntary and there was no cost to patients.

Our findings align with previous patient survey studies in prehabilitation which reported rates of at least 70% of patient participants expressing interest in prehabilitation regardless of prior knowledge of prehabilitation programs [28–30]. These studies also concluded that both education and personalization of multimodal programs to individual circumstances (e.g., finances, preference for home-based programs, baseline fitness) are essential to optimize patient engagement and therefore clinical outcomes. This emphasis on education and personalization aligns with the open-ended participant responses obtained in this study. These findings support future improvements in education and patient involvement to optimize personalized prehabilitation programs.

### Strengths and weaknesses of the study

A major strength of our study was the large representative sample of both cancer patients and carers at a large referral hospital including patients from metropolitan, regional/rural, and remote areas. This allows translation of our study to the wider cancer patient and carer population despite being a single-center study. The involvement of patients and carers is another strength of this research. Our consumer-centered study supports the relevance and usefulness of research priorities to the target end-users. Another strength was that the list of research priorities used was derived from an international, multidisciplinary group of clinicians with expertise in prehabilitation through the rigorous application of the Delphi technique. This meant the research priorities used in our study had been reviewed by several experts and are currently relevant in the context of emerging evidence. Another strength was that recall bias was reduced by recruiting patients within 4 weeks following surgery. This supported patient recall of the preoperative period and facilitated responses more relevant to postoperative recovery.

Despite these strengths, this study was not without weaknesses. While significant effort was made to be as inclusive as possible, this study may not necessarily represent the views of those who had inadequate English language competency to complete the survey. To maximize inclusivity and cultural competency, future studies will be conducted in collaboration with professionals who are competent in languages other than English. This would facilitate the inclusion of patients with language backgrounds and demographics not represented in this study. Additionally, as with other cohort studies, the characteristics of responders and non-responders could not be compared. Furthermore, patients and carers were potentially biased by the predefined list of 12 research priorities. To overcome this, the survey included open-ended questions to identify areas of importance to patients that were not considered by the clinician experts. Another weakness is that participant responses were potentially influenced by socioeconomic status and education level. Participants with limited health literacy and understanding of health research practicalities and implications were potentially less able to provide accurate answers. However, our sample is representative of a diverse surgical population at a public hospital. Furthermore, surveying patients and carers within 1 month following surgery minimized recall bias; however, the proposed longer-term benefits of prehabilitation (e.g., return to work, disease-free survival) were unlikely to be front of mind at this stage. Priorities may change over time. It is therefore important that long-term studies involving surveys at different time points, for example, preoperatively and then 6 months following surgery, are performed to identify changes in priorities over time. Additionally, further information could be provided if participants were compelled to rank research priorities from one to 12 in comparison to selecting importance on a scale from very high to very low. The former will be adopted in future studies to assess how the data would change. Furthermore, additional information regarding carers would be useful, particularly the proportion of carers who had direct experience of prehabilitation and the potential influence on their views. To achieve this, both carers and patients will be asked about their experience with prehabilitation in future studies.

### Strengths and weaknesses in relation to other studies

To our knowledge, this is the first study aiming to identify prehabilitation research priorities that included a broad population of multidisciplinary clinicians with expertise in prehabilitation, patients, and carers and included research priorities for multimodal prehabilitation while previous studies included only an expert group [18, 31] and focussed only on single modality of prehabilitation with exercise [31].

A published protocol by Pearson et al. [32] also detailed the use of the Delphi methodology to identify the top prehabilitation research priorities of patients, carers, and experts. It differs to our study as it includes both cancer and non-cancer patients and the expert group included colorectal surgeons only. A strength of Pearson’s study was the recruitment of patients from professional organizations and social media, enabling international perspectives; however, this is potentially also a weakness as patients engaging with social media and professional organizations may not represent the views of those who do not.

## Conclusion

The consensus of patient, carer, and expert views supports the direction of future high-quality research towards establishing the effectiveness of prehabilitation on surgical, functional, and patient-reported outcomes, the optimal composition of prehabilitation programs, and the safety and benefit of prehabilitation during neoadjuvant therapies. Research in other areas of prehabilitation remains important but perhaps only if required to address the current top priorities according to patients, carers, and experts, until these areas become well established.

### Supplementary Information

Below is the link to the electronic supplementary material.

Supplementary file1 (DOCX 19 KB)

## Data Availability

Available upon request.
