# Supplementary material for: Patients’ and carers’ views on research priorities in prehabilitation for cancer surgery
Source: Support Care Cancer. 2024 May 24;32(6):378. doi: 10.1007/s00520-024-08585-1 (PMC11126464; doi:10.1007/s00520-024-08585-1)
Supplement: Supplementary file 1 — Supplementary file1 (DOCX 19 KB) [file 520_2024_8585_MOESM1_ESM.docx]

Supplementary information

| **Supplementary Table 1.** Prehabilitation research priorities according to experts’, patients’, and carers’ views | | | | | | | | |
| --- | --- | --- | --- | --- | --- | --- | --- | --- |
| **Top research priorities** | **Rated as high or very high priority** | | | | **Median Score (IQR)** | | | |
|  | **Patients**  **(n=101)** | **Carers**  **(n=50)** | **Experts (n=121)** | p-value | **Patients**  **(n=101)** | **Carers**  **(n=50)** | **Experts**  **(n=121)** | p-value |
| **Effect of prehabilitation on surgical outcomes^** | 86 (85) | 45 (90) | 115 (95) | 0.044***** | 1 (1-2) | 1 (1-2) | 1 (1-2) | 0.371 |
| **Effect of prehabilitation on functional outcomes^** | 84 (83) | 43 (86) | 96 (79) | 0.545 | 2 (1-2) | 2 (1-2) | 2 (1-2) | 0.907 |
| **Prehabilitation during neoadjuvant therapies** | 82 (81) | 34 (68) | 83 (69) | 0.059 | 2 (1-2) | 2 (1-3) | 2 (2-3) | 0.001* |
| **Effect of prehabilitation on patient reported outcomes^** | 79 (78) | 42 (84) | 95 (79) | 0.673 | 2 (1-2) | 1 (1-2) | 1.5 (1-2) | 0.513 |
| **Optimal composition of prehabilitation programs^** | 78 (77) | 41 (82) | 107 (88) | 0.083 | 2 (1-2) | 2 (1-2) | 1 (1-2) | 0.586 |
| **Optimal nutritional regimen** | 73 (72) | 41 (82) | 82 (68) | 0.168 | 2 (1-3) | 2 (1-2) | 2 (1-3) | 0.019***** |
| **Enhancing compliance and adherence** | 73 (72) | 35 (70) | 91 (75) | 0.759 | 2 (1-3) | 2 (1-3) | 2 (1-2) | 0.945 |
| **Identifying populations most likely to benefit from prehabilitation** | 71 (70) | 38 (76) | 109 (90) | <0.001***** | 2 (1-3) | 2 (1-2) | 1 (1-2) | <0.001***** |
| **Screening tools to identify patients for consideration of prehabilitation** | 70 (69) | 38 (76) | 83 (69) | 0.609 | 2 (1-3) | 2 (1-2) | 2 (1-3) | 0.827 |
| **Modes of delivery for prehabilitation** | 69 (68) | 36 (72) | 84 (69) | 0.898 | 2 (1-3) | 2 (1-3) | 2 (2-3) | 0.366 |
| **Defining prehabilitation core outcome measures** | 67 (66) | 37 (74) | 105 (87) | 0.004***** | 2 (2-3) | 2 (1-3) | 2 (1-2) | 0.008***** |
| **Cost effectiveness of prehabilitation programs** | 62 (61) | 27 (54) | 92 (76) | 0.008***** | 2 (1-3) | 2 (1-3) | 2 (1-2) | 0.124 |
| Data presented as frequency (percentage). Research priorities listed in descending order from highest priority to lowest priority according to patient consensus. Research priority rating scale: 1= very high priority, 2= high priority, 3= moderate priority, 4=low priority, or 5=very low priority. High research priority determined by the number of experts, patients and carers that rated 1 (very high priority) or 2 (high priority) in the research priority rating scale. Research priority reached consensus of high importance by a group if rated as 1 (very high) or 2 (high) by >70% of participants in the group. ^Research priority reached consensus of high importance by all groups. *Significant difference between ratings across expert, patient, and carer groups (p<0.05). | | | | | | | | |
